# Development and In Vitro Evaluation of Linear PEI-Shelled Heparin/Berberine Nanoparticles in Human Osteosarcoma U-2 OS Cells

**DOI:** 10.3390/molecules23123121

**Published:** 2018-11-28

**Authors:** Hung-Kun Hsu, Kuang-Hsing Hsu, Ya-Ming Cheng, Hao-Yi Suen, Shu-Fen Peng

**Affiliations:** 1 Department of Chemistry, National Tsing Hua University, 30013 Hsinchu, Taiwan; d947624@gmail.com; 2 Department of Biological Science and Technology, China Medical University, 40402 Taichung, Taiwan; s5439003@hotmail.com (K.-H.H.), i04304v@gmail.com (H.-Y.S.); 3 Department of Agronomy, National Chung Hsing University, 40227 Taichung, Taiwan; ymcheng@dragon.nchu.edu.tw; 4 Department of Medical Research, China Medical University Hospital, 40402 Taichung, Taiwan

**Keywords:** Berberine, linear PEI, heparin, osteosarcoma, apoptosis

## Abstract

Berberine (BBR), a natural isoquinoline alkaloid derived from Chinese herbs, exerts many biological effects, including antiviral, antimicrobial, antidiarrhea, anti-inflammatory, and antitumor effects. In this study, a novel berberine nanoparticle (NP) consisting of heparin (HP) and BBR with or without being shelled with linear polyethyleneimine (LPEI) was developed to enhance its antitumor activity on osteosarcoma U-2 OS cells. With varying ratios of HP to BBR, HP/BBR NPs had a size ranging from 218.4 ± 3.9 to 282.0 ± 5.1 nm and zeta potential from −35.7 ± 0.4 to −51.9 ± 1.8 mV. After shelling with LPEI, the resultant NPs (HP/BBR/LPEI) possessed a size ranging from 226.3 ± 3.0 to 405.7 ± 85.2 nm and zeta potential from −46.5 ± 0.3 to −35.6 ± 0.5 mV; the encapsulation rate of BBR was close to 80%. The release profiles of both NPs were revealed to be slower than that of BBR solution. Results also showed that BBR and its two derived NPs reduced the viability of U-2 OS cells, and BBR NPs increased the cellular uptake of BBR. Cells were arrested at the G_1_ phase when treated individually with BBR and the two NPs (HP/BBR and HP/BBR/LPEI) and DNA condensation was induced. In addition, BBR and BBR NPs reduced the expression of mouse double minute 2 homolog (MDM2) but increased that of p53, and BBR NPs enhanced apoptotic effects. In short, heparin-based nanoparticles could be potential carriers for osteosarcoma treatment.

## 1. Introduction

Berberine (BBR) is a natural isoquinoline alkaloid derived from Chinese herb plants including *Coptis chinensis*, *Berberis aquifolium*, *Berberis vulgaris*, and *Coscinium fenestratum* [1]. It has been shown to have multiple pharmacological effects such as antimicrobial [2], antidiabetic [3], and cholesterol-lowering effects [4]. Recently, BBR has attracted a lot of attention due to its anticancer effects on many human cancer cells including osteosarcoma, leukemia, lung cancer, melanoma, colon cancer, and prostate cancer [5,6,7,8,9]. BBR has been shown to inhibit the proliferation of cancer cells [10,11] and HER2-overexpressing breast cancer cells [12] and to induce cell cycle arrest [13,14] and apoptosis [15,16].

The clinical applications of BBR are handicapped by its poor absorption and bioavailability. After oral administration, BBR is poorly absorbed in the gastrointestinal tract, resulting in poor bioavailability [1,17,18]. To overcome this shortcoming, nanoparticle-mediated delivery systems have been developed for new therapeutic strategies [19]. BBR nanoparticles have improved the solubility and enhanced the bioavailability of the drug, prolonged the circulation time, and reduced its side effects [20,21].

BBR-loaded nanoparticles enhanced the therapeutic effects of BBR on cancer cells. When compared with berberine solution, BBR-loaded solid lipid nanoparticles (SLNs) significantly inhibited proliferation in MCF 7, Hep G2, and A549 cancer cells. In addition, BBR-loaded SLNs increased cellular uptake of BBR, decreased colony formation of cancer cells, and induced cell apoptosis in MCF 7 cancer cells [19]. O-hexadecyl-dextran entrapped BBR nanoparticles were shown to be as effective as BBR solution at a 20-fold lower concentration in preventing oxidative stress, mitochondrial depolarization, and downstream events of apoptotic cell death in high-glucose-stressed primary hepatocytes [22]. Silica nanoconjugates bearing a covalently linked BBR enhanced apoptosis cell death in a human cervical carcinoma cell line (HeLa), a human hepatocellular carcinoma cell line (Hep G2), and human embryonic kidney (HEK) 293T cell lines when compared with cells treated with BBR [23].

Sulfate-containing polymers have been used in complex with the positively charged BBR for different applications. BBR-loaded heparin (HP) nanoparticles significantly suppressed the growth of *Helicobacter pylori (H. pylori)* and efficiently reduced cytotoxic effects on *H. pylori*-infected cells [24]. BBR-loaded chitosan/fucoidan–taurine nanoparticles were found to be effective for the treatment of a defective intestinal barrier caused by bacterial endotoxin [25]. Fucoidan–taurine conjugation (sulfonated fucoidan) was employed complexed with BBR to target intestinal epithelial cells and then release BBR to restore the injured cells.

In this study, we develop a promising polyethyleneimine (PEI)-shelled nanoparticle for BBR delivery to induce osteosarcoma cell apoptosis. The characteristics of the BBR nanoparticles (HP/BBR or HP/BBR/LPEI nanoparticles) were examined by dynamic light scattering (DLS), the internalization efficiency by fluorescence microscopy and flow cytometry, and the cell cycle distributions by flow cytometry. Finally, the apoptotic effects of BBR-loaded nanoparticles on U-2 OS cells were evaluated by 4’,6-diamidino-2-phenylindole (DAPI) staining and Western blotting.

## 2. Results

### 2.1. Characterizations of Heparin/Berberine (HP/BBR) and Heparin/Berberine/Linear Polyethylenimine (HP/BBR/LPEI) Nanoparticles

**Figure 1:**
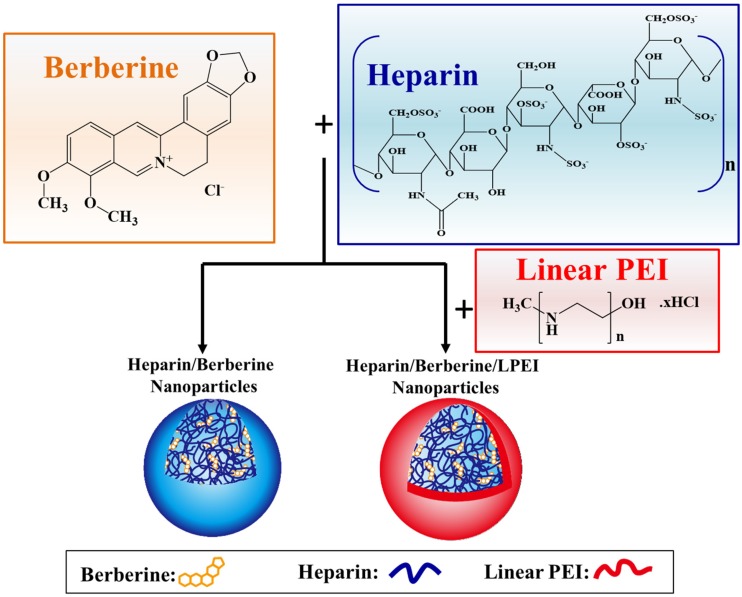
A schematic diagram of heparin (HP)/berberine (BBR) or HP/BBR/linear polyethyleneimine (LPEI) nanoparticles and their preparation. The nanoparticles were prepared at various weight ratios of HP/BBR/LPEI by electrostatic interaction. First, different amounts of heparin were complexed with a defined amount of BBR to form HP/BBR nanoparticles by sonicating. Second, the HP/BBR nanoparticles were coated with various amounts of LPEI solution to result in HP/BBR/LPEI nanoparticles by thoroughly mixing. Abbreviation: BBR, berberine; HP, heparin; LPEI, linear polyethylenimine.

Anionic heparin (HP) containing sulfate groups showed excellent binding capacity with cationic berberine (BBR) to result in HP/BBR nanoparticles (HP/BBR NPs). Furthermore, positively charged linear PEI (LPEI) shelled negatively charged HP/BBR NPs to form HP/BBR/LPEI NPs (Figure 1). The HP/BBR/LPEI NPs were designed to decrease the rate of drug release and enhance cellular uptake and apoptosis effects.

The nanoparticles (HP/BBR or HP/BBR/LPEI) were characterized by several properties including size, zeta potential, and encapsulation efficiency. As shown in Table 1, HP/BBR NPs prepared with various weight ratios (24/100, 48/100, 72/100, 96/100, 120/100, or 144/100) had various sizes ranging from 218.4 ± 3.9 to 282.0 ± 5.1 nm in diameter, revealing a narrow size distribution with a small polydispersity index (PDI; 0.17 ± 0.001 to 0.20 ± 0.02). When prepared at weight ratios of 24/100 to 144/100, the nanoparticle’s zeta potential possessed a net negative charge on its surface. Increasing the HP content from 24 to 144 μg resulted in decrease of zeta potential (−35.7 ± 0.4 to −51.9 ± 1.8 mV) owing to more HP carrying large amounts of sulfate or carboxyl groups (Figure 1 and Table 1). The BBR loading efficiencies at various weight ratios of HP/BBR ranged from 49.9 ± 1.3 to 91.4 ± 0.4%, indicating a high binding capacity between BBR and HP. The NPs prepared to the weight ratio of 48/100 exhibited an encapsulation efficiency of 91 ± 0.4%, and those prepared to the 72/100 ratio, had an encapsulation efficiency of 78.3%. Small nanoparticles with high encapsulation efficiency were selected for the following preparation for coating with LPEI.

Shelling LPEI on the surface of HP/BBR NPs changed the characteristics of the nanoparticles (Table 2). With an increase in LPEI amount (0–16 μg), HP/BBR/LPEI NPs (72/100/0 to 72/100/16) had increased diameters (226.3 ± 3.0 to 405.7 ± 85.2 nm), and their zeta potentials were increased as well (−46.5 ± 0.3 to −35.6 ± 0.5 mV) as a result of more positively charged LPEI coated on their surface. After coating with LPEI, the resulting NPs (HP/BBR/LPEI) showed an increase in PDI (from 0.19 ± 0.01 to 0.78 ± 0.02), indicating the heterogenous distribution of NPs after a higher amount of LPEI coating. The encapsulation efficiency did not change after LPEI coating. Based on the results of Table 1 and Table 2, HP/BBR and HP/BBR/LPEI NPs at weight ratios of 72/100 and 72/100/8, respectively, were used in the following experiments.

### 2.2. The Release Profiles of Berberine and Berberine Nanoparticles

**Figure 2:**
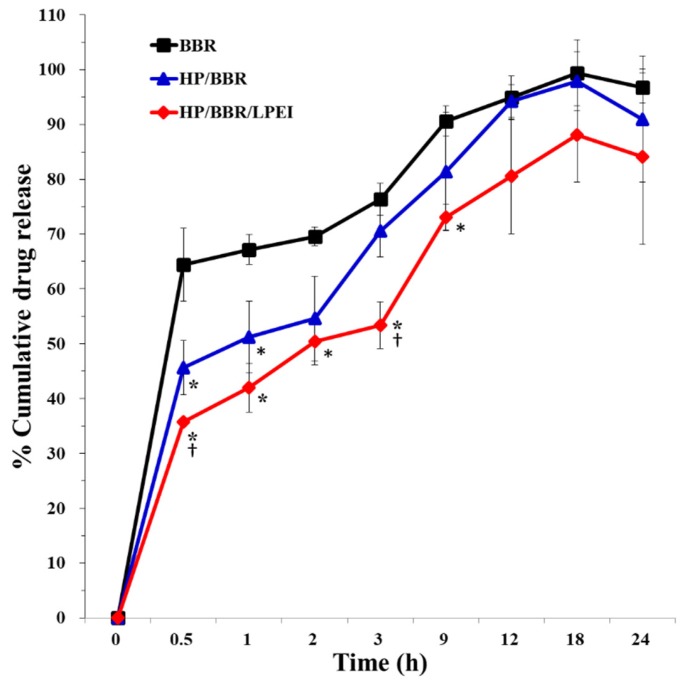
The release profiles of BBR solution and BBR NPs (HP/BBR or HP/BBR/LPEI NPs) for 24 h. * *p* < 0.05 significant difference between BBR-treated groups and the control and † *p* < 0.05 significant difference between HP/BBR-treated groups and HP/BBR/LPEI groups as analyzed by one-way ANOVA with post hoc Tukey HSD (Honest Significant Difference).

The release profiles of BBR from nanoparticles were investigated in a simulated physical environment (pH 7.4 for 24 h). BBR and BBR NPs (HP/BBR and HP/BBR/LPEI NPs) were loaded into dialysis membranes (with pore sizes of 6–8000 Da) which were filled with PBS to investigate the release profiles of BBR from the BBR solution and BBR NPs. As shown in Figure 2, the BBR release rates of HP/BBR/LPEI NPs were significantly slower than those of BBR solution and HP/BBR NPs due to the LPEI shell coating on the surface of the nanoparticles.

### 2.3. The Effects of Berberine and Berberine Nanoparticles on Cell Viability of Osteosarcoma U-2 OS Cells

**Figure 3:**
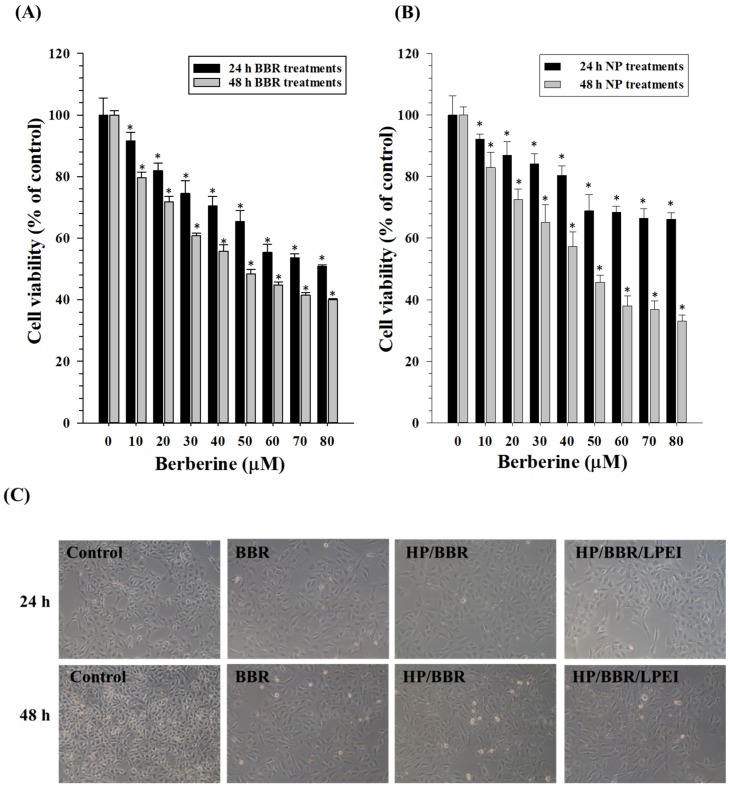
The cell viability and morphology of U-2 OS cells treated with BBR or BBR nanoparticles (HP/BBR or HP/BBR/LPEI nanoparticles). (**A**) U-2 OS cells were treated with different concentrations (0, 10, 20, 30, 40,50, 60, 70, and 80 μM) of BBR for 24 or 48 h. (**B**) U-2 OS cells were treated with HP/BBR/LPEI nanoparticles containing different concentrations (0, 10, 20, 30, 40,50, 60, 70, and 80 μM) of BBR for 24 or 48 h. (**C**) The morphology of U-2 OS cells treated with 50 μM BBR, or HP/BBR or HP/BBR/LPEI nanoparticles containing 50 μM BBR for 24 or 48 h. Cells without any treatment were used as a control group. *p < 0.05 significant difference between BBR-treated groups and the control as analyzed by one-way ANOVA.

To assess the cytotoxic effects of BBR on human osteosarcoma U-2 OS cells, cells were treated with various doses of BBR (0, 10, 20, 30, 40, 50, 60, 70, and 80 μM) for 24 or 48 h, and then the cell viability was analyzed by 3-(4,5-Dimethylthiazol-2-yl)-2,5-diphenyltetrazolium bromide (MTT) assay. As shown in Figure 3A, the viable cell number of U-2 OS cells was decreased by BBR in a dose-dependent manner. Furthermore, cells treated with various concentration of HP/BBR/LPEI NPs with BBR concentrations of 0, 10, 20, 30, 40, 50, 60, 70, and 80 μM, also showed a similar decrease in viable cell number. However, the cytotoxicity of HP/BBR/LPEI NPs was less efficient than that of BBR solution at 24 h after treatment. After 48 h, cells treated with HP/BBR/LPEI NPs significantly reduced the viable cell number when compared with the control group without any treatment (Figure 3B). This finding suggests that BBR and HP/BBR/LPEI NPs have antitumor activities, and the effect of HP/BBR/LPEI NP is more pronounced at 48 h after treatment.

After exposure to BBR or BBR NPs containing 50 μM of BBR, morphological changes associated with U-2 OS cells were visualized by microscopy after either 24 or 48 h (Figure 3C). The viable cell number was obviously lower in the treatment with HP/BBR/LPEI NPs than in either the treatment with the BBR solution or the control group.

### 2.4. The Cellular Uptake of Berberine and Berberine Nanoparticles

**Figure 4:**
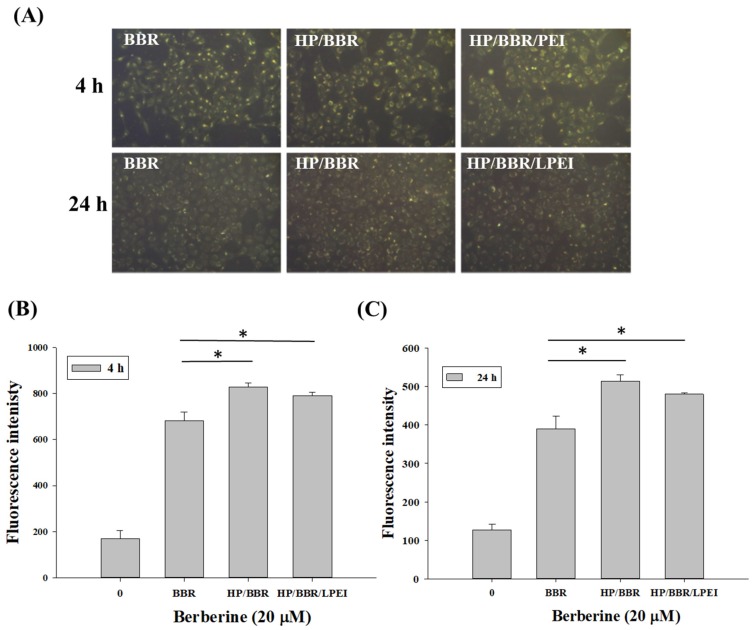
Cellular uptake of BBR, HP/BBR nanoparticles, and HP/BBR/LPEI nanoparticles containing 20 μM BBR in U-2 OS cells after 4 or 24 h treatments. (**A**) The uptake of BBR, HP/BBR nanoparticles, and HP/BBR/LPEI nanoparticles in U-2 OS cells measured by flow cytometry after 4 h (**B**) and 24 h (**C**) of treatment. *p < 0.05 significant difference between BBR-treated groups and the control as analyzed by one-way ANOVA.

Cellular uptake of BBR and BBR NPs in U-2 OS cells was presented in images and quantified by fluorescent microscopy and flow cytometry, respectively. As shown in Figure 4A,B, the uptake amounts of HP/BBR and HP/BBR/LPEI NPs after 4 h of treatment were higher than that of BBR, indicating an enhanced uptake of the BBR NPs (*p* < 0.05). After 24 h of treatment, the cells treated with BBR NPs had higher BBR intensity than did those treated with BBR (*p* < 0.05) (Figure 4A,C). This suggests that the nanoparticle form of BBR will enhance the uptake amount and prolong the retention time of BBR in U-2 OS cells.

### 2.5. Cell Cycle Distribution of U-2 OS Cells Treated With Berberine and Berberine Nanoparticles

**Figure 5:**
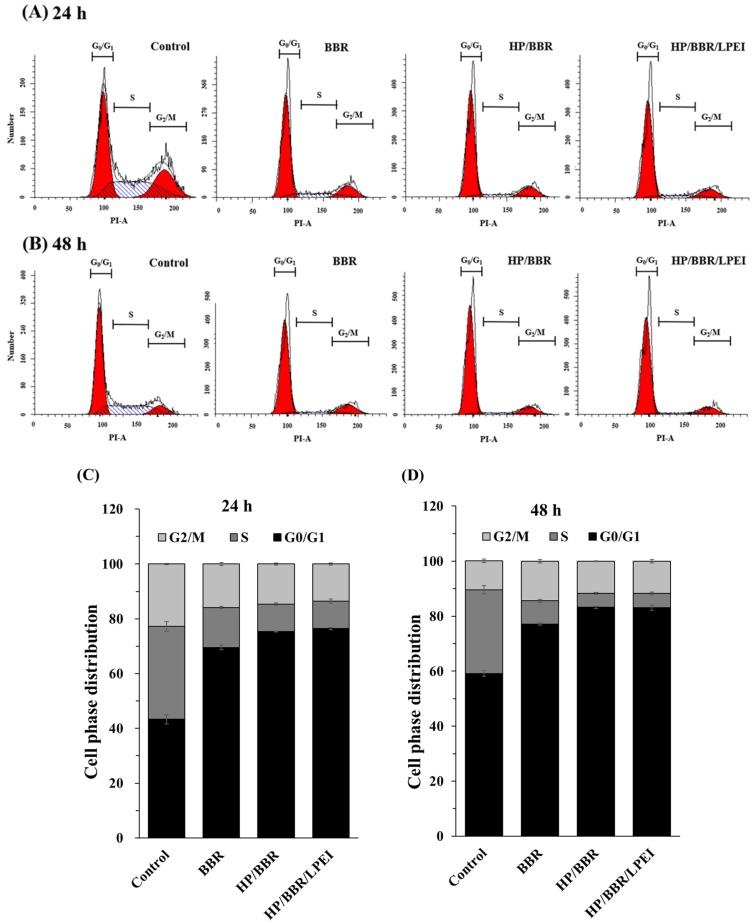
The effects of BBR and BBR NPs (HP/BBR and HP/BBR/LPEI NPs) on cell cycle distribution in U-2 OS cells. U-2 OS cells were treated with BBR, HP/BBR NPs, or HP/BBR/LPEI NPs containing 50 μM BBR for 24 or 48 h and were harvested for cell cycle distribution assay by flow cytometry assay. The results of the flow cytometry assays after 24 h (**A**) and 48 h (**B**) are presented, and the cell cycle distributions in percentages after 24 h (**C**) and 48 h (**D**) of treatment are presented. Experiments were performed in triplicate as described in Materials and Methods.

To investigate whether the decrease in the growth rate of cells treated with BBR and BBR NPs was associated with cell cycle arrest, we analyzed propidium iodide (PI)-stained U-2 OS cells treated with BBR solution and BBR NPs (HP/BBR NPs and HP/BBR/LPEI NPs) containing 50 μM BBR for 24 or 48 h by flow cytometry. As shown in Figure 5A,B, after treatment with BBR, HP/BBR NPs, or HP/BBR/LPEI NPs, U-2 OS cells displayed significant accumulation of the G_0_/G_1_ phase as compared to the control group, indicating that both BBR and BBR NPs exerted effects of cell cycle arrest at the G_0_/G_1_ phase in U-2 OS cells.

### 2.6. BBR and BBR NPs Induce DNA Condensation

**Figure 6:**
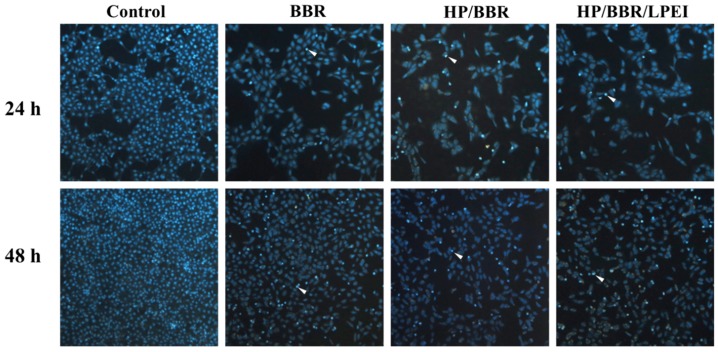
BBR and BBR NPs (HP/BBR and HP/BBR/LPEI NPs) induce nuclear chromatin condensation in U-2 OS cells. Cells were treated with BBR, HP/BBR NPs, and HP/BBR/LPEI NPs containing 50 μM BBR for 24 or 48 h; then, cells were stained with 4’,6-diamidino-2-phenylindole (DAPI) as described in Materials and Methods. Cells were examined and photographed using a fluorescence microscope at 100×. Arrowheads show the representative DNA condensation.

To determine whether BBR and BBR NPs (HP/BBR and HP/BBR/LPEI NPs) could induce DNA damage and condensation, U-2 OS cells were treated with BBR and BBR NPs (HP/BBR and HP/BBR/LPEI) containing 50 μM BBR for 24 or 48 h, stained with DAPI, and photographed by fluorescence microscopy. As shown in Figure 6, HP/BBR and HP/BBR/LPEI NPs induced brighter DAPI fluorescence in the nuclei of U-2 OS cells after 24 and 48 h of treatment in comparison with the control group, indicating that the DNA of the cells has been nicked and their nuclear chromatin has been condensed.

### 2.7. Berberine and Berberine Nanoparticles Induce the Expression of p53 and Apoptosis-Associated Proteins

**Figure 7:**
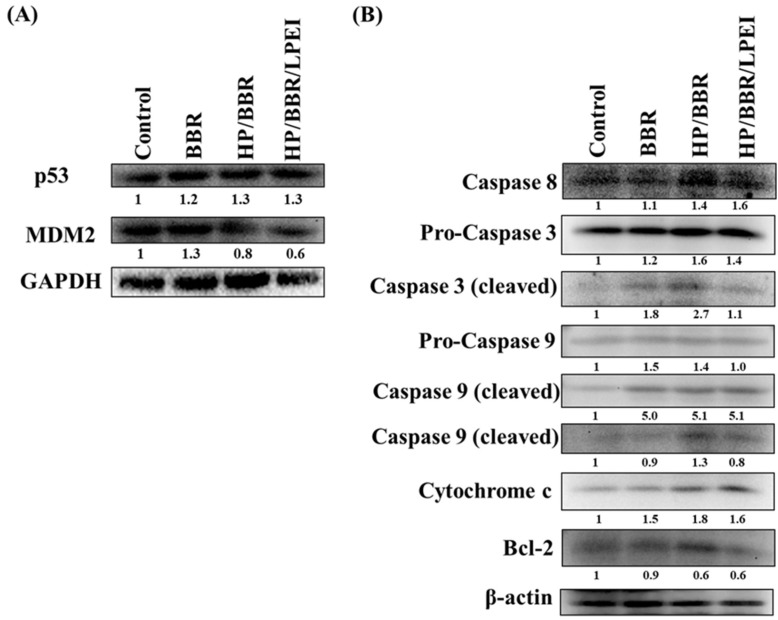
BBR affects the level of p53 and apoptosis-associated proteins in U-2 OS cells. (**A**). Cells were treated with BBR, HP/BBR NPs, and HP/BBR/LPEI NPs containing 50 μM BBR for 48 h. Cells were collected and total proteins were analyzed by Western blotting. The levels of p53 and mouse double minute 2 homolog (MDM2) (**A**) and expressions of apoptosis-associated proteins including caspase 8, caspase 3, caspase 9, cytochrome c, and Bcl-2 (**B**) were determined by Western blotting as described in Materials and Methods.

To study the effects of BBR on the expression of p53 and apoptosis-associated proteins, cells were treated with BBR, HP/BBR NPs, and HP/BBR/LPEI NPs containing 50 μM of BBR for 48 h and harvested for Western blotting analysis. Results indicated that cells treated with BBR, HP/BBR NPs, and HP/BBR/LPEI NPs suppressed expression of mouse double minute 2 homolog (MDM2) but increased expression of p53 (Figure 7A). Furthermore, cells treated with BBR, HP/BBR NPs, and HP/BBR/LPEI NPs containing 50 μM BBR augmented the expression of caspase-8, cleaved caspase-3, cleaved caspase-9, and cytochrome c, but decreased that of Bcl-2 (Figure 7B). These observations indicate that BBR is capable of inducing apoptotic cell death, and such an effect is more pronounced in BBR NPs.

## 3. Discussion

Berberine (BBR) induces anticancer effects in many cancer cell lines including osteosarcoma. It is a quaternary alkaloid with a positively charged amine. In previous study, we successfully prepared BBR nanoparticles (BBR NPs) using negatively charged heparin (HP) in complex with BBR for an antibacterial study. In this study, we found that BBR suppressed the proliferation and viability of osteosarcoma cells through induction of cell cycle arrest and apoptosis. Herein, we used HP to complex with BBR, and the resultant nanoparticles were coated with linear polyethylenimine (LPEI) to form novel BBR NPs. The HP/BBR/LPEI NPs were used to suppress cell viability and to induce apoptosis in U-2 OS cells.

Due to the isoquinoline structure, BBR shows poor solubility in water and low bioavailability. The solubility problem can be improved by its nanoparticle form, and the bioavailability can be enhanced by complexing with HP. HP bearing negatively charged groups was used to complex with positively charged BBR [24,25], and the resultant nanoparticles were coated with linear PEI (LPEI) to result in the HP/BBR/LPEI NPs used in this study. As shown in Figure 2, HP/BBR and HP/BBR/LPEI NPs changed the release profile of BBR in PBS buffer (pH 7.4).

HP, an anticoagulant and a kind of polysaccharide, is a highly heterogeneous sulfated glycosaminoglycan [26]. It and its derivatives, components of the extracellular matrix (ECM), play cell signaling roles [27]. In addition, it has been utilized as carrier for the delivery of doxorubicin and hydrophobic lappaconitine to enhance their drug effects [28,29]. The PEI used in this study is a linear polymer, designated linear PEI (LPEI). In a physiological pH environment, positively charged LPEI and negatively charged HP were able to form polyplexes via electrostatic interaction. In this study, LPEI was coated on the surface of HP/BBR NPs to reduce the release rate of BBR (Figure 2).

BBR NPs increased drug solubility in water and enhanced bioavailability, resulting in improved pharmacological activity [30]. Nanoparticles encapsulating BBR enhanced the cellular uptake of BBR (Figure 4), induced cell cycle arrest at the G_0_/G_1_ phase at 24 and 48 h (Figure 5), and enhanced cell apoptosis (Figure 7B). The literature has reported that BBR has induced cancer cell arrest at the G_0_/G_1_ phase for human osteosarcoma, prostate, colon, and bladder cancer cells [5,7,14,15]. Our results confirmed that BBR NPs (HP/BBR and HP/BBR/LPEI) enhanced BBR’s effects on cell cycle arrest (Figure 5) in U-2 OS cells, indicating that the encapsulated BBR still maintains its biological function and can be released to cells from nanoparticles. The DAPI staining results (Figure 6) revealed that BBR and BBR NP treatments invoked chromatin condensation in U-2 OS cells, indicating an induction of DNA damage.

Inactivation or mutation of the p53 gene and shutdown of the p53 protein are key processes in tumor development [31,32]. p53 plays multiple tumor suppressive roles in cells, inducing damage stresses and regulation of G_1_ and G_2_ arrest of the cell cycle or apoptosis, depending on the cell type. BBR was shown to induce growth inhibition of non-small cell lung cancer cells through p53 regulation [33]. The literature has reported that cells carrying wild-type (wt) p53 seem more susceptible to BBR. LNCaP cells carrying wild-type (wt) p53 were more susceptible to BBR than PC-3 cells lacking p53 after 48 h of treatment [7]. However, the osteosarcoma U-2 OS and Saos-2 cells, which express or lack p53, respectively, displayed similar IC_50_ concentrations (20 μg/mL) of BBR over a 48 h treatment [5]. This may due to the fact that U-2 OS cells carry wt p53 and its MDM2 is overexpressed, leading to low p53 expression [14]. This supposition was confirmed in this study. We designed BBR NPs and used them to treat U-2 OS cells, and we found that after 48 h of treatment, MDM2 expression was diminished but p53 expression was enhanced (Figure 7A).

The nanoparticle form of BBR showed here was effective in inducing apoptosis in U-2 OS cells. Expressions of caspase 8, cleaved caspase 3, cleaved caspase 9, and cytochrome c were greatly enhanced. However, anti-apoptotic Bcl-2 was decreased after 48 h of BBR treatment (Figure 7A). BBR NPs may be a potential treatment for cancer cells carrying the wt p53 genotype with overexpressed MDM2.

In this report, we successfully used HP to complex with BBR, and then coated the resulting particles with positively charged LPEI to result in HP/BBR/LPEI NPs. The HP/BBR/LPEI complex provides prolonged BBR retention inside cells and enhanced biological activities in apoptosis induction. The long-term effects of this complex will need to be evaluated in the future. The abovementioned results indicate that the HP-based BBR nanoparticle can serve as a drug delivery vehicle for disease treatment.

## 4. Materials and Methods

### 4.1. Chemicals and Reagents

Berberine hydrochloride (BBR), linear PEI (LPEI, MW 25 kDa), MTT [3-(4,5-dimethylhiazol-2-yl)-2,5-diphenyltetrazolium bromide], dimethyl sulfoxide (DMSO), DAPI, propidium iodide, and McCoy’s *5A* medium were purchased from Sigma-Aldrich (St. Louis, MO, USA). Heparin (HP) was purchased from China Chemical and Pharmaceutical Co., Ltd (Taipei, Taiwan). Fetal bovine serum (FBS), L-glutamine, and penicillin–streptomycin were purchased from GIBCO^®^/Invitrogen Life Technologies (Carlsbad, CA, USA). Primary antibodies against caspase 3, caspase 8, Bcl-2, and p53 were purchased from GeneTex Inc. (Irvine, CA, USA); MDM2 was purchased from Cell Signaling Technology, Inc. (Beverly, MA, USA); and caspase 9, glyceraldehyde-3-phosphate dehydrogenase (GAPDH), β-actin, and horseradish peroxidase (HRP)-conjugated secondary antibodies were purchased from Novus Biologicals (Littleton, CO, USA).

### 4.2. Preparation of HP/BBR or HP/BBR/LPEI Nanoparticles

The nanoparticle preparations were based on the weight ratios of materials used in this study. The weight ratio of nanoparticles was expressed as the weight ratio of the negatively charged HP to the positively charged BBR or LPEI (HP/BBR or HP/BBR/LPEI NPs, respectively). NPs were prepared at various weight ratios of HP/BBR (24/100, 48/100, 72/100, 96/100, 120/100, or 144/100) by electrostatic interaction as illustrated in Figure 1. Briefly, different amounts of heparin (24, 48, 72, 96, 120, or 108 μg) were complexed with a defined amount (100 μg) of BBR to bring these solutions to a total volume of 900 μl by sonicating for 60 s. HP/BBR NPs at a weight ratio of 72/100 were selected for preparing HP/BBR/LPEI NPs in two separated steps. First, HP/BBR NPs were prepared by mixing 72 μg HP and 100 μg BBR solution by sonicating for 60 s. Second, various amounts of LPEI solution (0, 4, 8, 12, or 16 μg) were added to the HP/BBR NP solutions by thoroughly mixing for 30 s, and then leaving the solution for at least 1 h at room temperature.

### 4.3. Particle Size and Zeta Potential Measurements

The size distributions and zeta potentials of HP/BBR or HP/BBR/LPEI NPs were measured by using a Zetasizer Nano ZS (Malvern Instruments Ltd., Worcestershire, UK).

### 4.4. Encapsulation Efficiency Assay of BBR

The nanoparticles prepared at various weight ratios were examined for their encapsulation efficiencies. Nanoparticles prepared at various weight ratios were centrifuged at 13,000 rpm for 30 min, and the supernatants containing unencapsulated BBR were transferred to new microtubes for concentration determination. A known concentration of BBR solution was used as a standard. The collected supernatants of NPs prepared under different experimental conditions were transferred to a 96-well plate, and the absorption wave of OD_420 nm_ was measured by using an ELISA reader (TECAN, Männedorf, Switzerland). The BBR in the supernatant was determined by comparing to the standard [34]. The encapsulation efficiency was calculated using Equation (1) as follows:

Encapsulation efficiency (%) = [(total BBR − BBR in supernatant)/total BBR] × 100.(1)

### 4.5. Release Profile

The release profiles of BBR from BBR solution or from prepared nanoparticles (HP/BBR or HP/BBR/LPEI) were investigated in PBS (pH 7.4) at 37 °C under agitation. BBR solutions (0.5 mL) including BBR, HP/BBR NPs, or HP/BBR/LPEI NPs were loaded into dialysis tubes (MW 6–8000 Da), and the dialysis tubes were immersed in 3 mL PBS solution in 15 mL centrifuge tubes. Fixed volumes (0.2 mL) of the released BBR solution were removed to a new microtube and refilled with 0.2 mL PBS to the 15 mL centrifuge tube. The collected supernatants were subjected to an ELISA reader to attain the absorption values of OD_420 nm_ which were compared with the standard curve of BBR. The percentage of cumulative BBR released from the dialysis tubes was determined using a standard calibration curve.

### 4.6. Cell Culture

Human osteosarcoma U-2 OS cells were obtained from the Food Industry Research and Development Institute (BCRC Number: 60187; Hsinchu, Taiwan). Cells were cultured in McCoy 5A medium supplemented with 2.0 g/L sodium bicarbonate, 100 units/mL penicillin, 100 μg/mL streptomycin, and 10% fetal bovine serum (FBS) in humidified air containing 5% CO_2_ at 37 °C.

### 4.7. Cell Viability Assay and Morphological Observation

The cell viability was assessed by MTT assay. The U-2 OS cells were cultured in a 96-well plate at the density of 5 × 10^3^ cells/well and were incubated with different concentrations of BBR (0, 10, 20, 30, 40, 50, 60, 70, and 80 μM) for 24 or 48 h. Then, phenol-red-free culture medium containing 500 μg/mL MTT was added to each well and incubated at 37 °C for another 4 h. After incubation, the supernatant was removed. The blue formazan crystals formed in viable U-2 OS cells were dissolved with DMSO, and the value of OD_570 nm_ was then measured with the ELISA reader. All experiments were performed in triplicate. The morphological changes of U-2 OS cells treated with BBR, HP/BBR, and HP/BBR/LPEI NPs were examined under a phase-contrast microscope [35].

### 4.8. Cellular Uptake of Berberine and Berberine Nanoparticles

The internalizations of BBR solution and BBR-loaded NPs (HP/BBR or HP/BBR/LPEI) were tracked and quantified by fluorescence microscope and flow cytometry, respectively. U-2 OS cells (2 × 10^5^) were seeded on 12-well plates and incubated overnight. Then, cells were treated with BBR solution or BBR NPs (HP/BBR or HP/BBR/LPEI) containing 20 μM of BBR for 4 or 24 h. Cells were observed and photographed under a fluorescence microscope (Carl Zeiss Optical, Chester, VA, USA) to estimate the amount of cellular uptake. Then, cells were washed with PBS twice, trypsinized, and transferred to FACS tubes. Subsequently, the uptake amount of BBR by U-2 OS cells was analyzed using a flow cytometer [35].

### 4.9. Cell Cycle Assay

The cell cycle distribution was analyzed by flow cytometry. U-2 OS cells were cultured in a 12-well plate at a density of 2 × 10^5^ cells/well and incubated with BBR solution or BBR NPs (HP/BBR, HP/BBR/LPEI) containing 50 μM BBR for 24 or 48 h. After treatment, the cells were collected, washed with PBS, fixed with 70% ethanol, and stored at −20 °C overnight. Then, cells were centrifuged and re-suspended in PBS containing PI (10 μg/mL) and RNase A (100 μg/mL) at room temperature for 30 min. Stained cells were analyzed using a FACSCanto flow cytometer (Becton-Dickinson, Franklin Lakes, NJ, USA). The percentage of cell cycle phases was determined by using ModFit software [36].

### 4.10. DAPI Staining

U-2 OS cells (2 × 10^5^ cells/well) in 12-well plates were incubated with 50 μM of BBR solution or BBR NPs (HP/BBR and HP/BBR/LPEI) for 24 or 48 h. Cells were washed with PBS and fixed with 4% paraformaldehyde for 15 min, and cells were then stained with DAPI (1 μg/mL) in PBS at 37 °C for 30 min and were observed and photographed under fluorescence microscopy [36].

### 4.11. Western Blotting

The U-2 OS cells (1 × 10^6^) were individually treated with 50 μM BBR solution and BBR NPs (HP/BBR and HP/BBR/PEI NPs) for 48 h. Cells were harvested, lysed, and total proteins were then quantified using Bradford reagent. Approximately 20 μg of proteins from each treatment were resolved on SDS–polyacrylamide gel electrophoresis (SDS-PAGE) and transferred to a Polyvinylidene fluoride (PVDF) membrane (Merck Millipore, Burlington, MA, USA). The transferred membranes were blocked with 2.5% bovine serum albumin (BSA) in 20 mM Tris-buffered saline/0.05% Tween-20 (TBST) (Sigma-Aldrich, St. Louis, MO, USA) for 1 h at room temperature, followed by probing with primary antibodies against p53, MDM2, caspase 3, caspase 8, caspase 9, cytochrome c, Bcl-2, β-actin, or GAPDH at 4 °C overnight. Then, blots were incubated with secondary antibodies conjugated with HRP for enhanced chemiluminescence detection. Anti-β-actin and -GAPDH were used as loading controls.

### 4.12. Statistical Analysis

Comparison between groups was analyzed using one-way ANOVA. All data are presented as a mean value with the standard deviation indicated (mean ± SD). Differences were considered to be statistically significant when the *p* values were less than 0.05.

## Figures and Tables

**Table 1:** Size, zeta potential, and loading efficiency of HP/BBR nanoparticles (NPs) (*n* = 3).

Weight Ratio	Size (nm)	Polydispersity Index (PDI)	Zeta Potential (mV)	Loading Efficiency (%)
HP/BBR = 24/100	282.0 ± 5.1	0.20 ± 0.02	−35.7 ± 0.4	49.9 ± 1.3
HP/BBR = 48/100	272.0 ± 5.7	0.18 ± 0.02	−40.7 ± 0.7	91.4 ± 0.4
HP/BBR = 72/100	231.1 ± 6.4	0.17 ± 0.01	−45.7 ± 0.2	78.3 ± 0.3
HP/BBR = 96/100	232.7 ± 1.9	0.17 ± 0.03	−48.0 ± 1.0	73.8 ± 0.3
HP/BBR = 120/100	218.4 ± 3.9	0.20 ± 0.01	−44.8 ± 0.6	73.4 ± 1.3
HP/BBR = 144/100	232.9 ± 11.9	0.19 ± 0.00	−51.9 ± 1.8	77.4 ± 0.3

**Table 2:** Size, zeta potential, and loading efficiency of HP/BBR/LPEI NPs (*n* = 3).

Weight Ratio	Size (nm)	Polydispersity Index (PDI)	Zeta Potential (mV)	Loading Efficiency (%)
HP/BBR/LPEI = 72/100/0	226.3 ± 3.0	0.19 ± 0.01	−46.5 ± 0.3	82.0 ± 0.4
HP/BBR/LPEI = 72/100/4	227.1 ± 4.2	0.22 ± 0.01	−44.9 ± 0.7	82.2 ± 1.7
HP/BBR/LPEI = 72/100/8	234.8 ± 6.3	0.32 ± 0.03	−41.9 ± 1.0	86.2 ± 0.3
HP/BBR/LPEI = 72/100/12	405.7 ± 85.2	0.56 ± 0.12	−36.1 ± 1.2	87.4 ± 1.8
HP/BBR/LPEI = 72/100/16	402.3 ± 10.4	0.78 ± 0.02	−35.6 ± 0.5	81.1 ± 0.8
